# Occupation and bladder cancer: a cohort study in Sweden

**DOI:** 10.1038/sj.bjc.6602473

**Published:** 2005-03-15

**Authors:** J Ji, C Granström, K Hemminki

**Affiliations:** 1 Department of Bioscience at Novum, Karolinska Institute, 141 57 Huddinge, Sweden; 2 Division of Molecular Genetic Epidemiology, German Cancer Research Center (DKFZ), Im Neuenheimer Feld 580, 69120 Heidelberg, Germany

**Keywords:** bladder cancer, occupation, follow-up study, epidemiology

## Abstract

In a follow-up study of occupational exposures and bladder cancer, an increased risk was observed after an adjustment for smoking, for physicians, administrators and managers, clerical workers and sales agents among men and assistant nurses among women. For physicians, the reason may be early diagnosis; for the other groups a sedentary type of work may have a role in bladder cancer aetiology.

Occupation has been identified as the second most important risk factor for bladder cancer after smoking and estimated to account for as much as 20% of all bladder cancer in industrialised countries (Vineis and Simonato, 1991). Despite considerable efforts to investigate occupation and industry in relation to bladder cancer risk, many reported associations have not been found consistently (Zahm *et al*, 1987; Carpenter and Roman, 1999; Simpson *et al*, 1999). Furthermore, occupational risks of bladder cancer have changed over time and differ from population to population, so there is a need to determine whether certain occupations are no longer at risk and possibly to identify new high-risk occupations (Silverman *et al*, 1989). We have therefore carried out a follow-up study on the economically active Swedish population, based on the Swedish Family-Cancer Database, which covered about 3.3 million men and 2.8 million women.

## MATERIALS AND METHODS

The Swedish Family-Cancer Database was created in the mid-1990s by linking an administrative family register on all Swedish families at Statistics Sweden to the Swedish Cancer Registry. Additionally, death notification data and data from four national censuses (1960, 1970, 1980, 1990) were included. The Database was updated in 2002 to include cancers from the Cancer Registry from 1961 to 2000 (Hemminki *et al*, 2001). A four-digit diagnosis code based on the 7th revision of the *International Classification of Diseases* (ICD-7) has been used since 1958. Only first primary bladder cancers (ICD 181.0) were considered in the present study. National census data conducted by Statistics Sweden included employment status, job title and work industry occupation being coded according to an adapted Nordic Occupational Classification, which defined a total of 53 occupational groups (Andersen *et al*, 1999).

Standardised incidence ratios (SIRs) were calculated as the ratio of the observed (O) to expected numbers of cases, the latter being obtained by applying incidence rates for men and women with occupation in either the 1960 or 1970 census, or with the same occupation in two consecutive censuses (1960 and 1970), or the same occupation in the three consecutive censuses (1960, 1970 and 1980). Various occupational groups should experience the same cancer incidence as in the corresponding economically active population in the Database, calculated from 5-year-age, period (10 years bands), socio-economic status (six groups). To allow for the confounding effect of smoking for occupations with significantly increased lung cancer risks, we also calculated a smoking corrected SIR, dividing the SIRs by 35% of the excess of lung cancer risk. The figure of 35% is based on the IARC (2004) summary of cancer risks for smokers of 20 cigarettes per day being 10–20 for lung cancer and five for bladder cancer. Confidence intervals (95% CIs) were calculated assuming a Poisson distribution. Follow-up was started at immigration or on 1 January following the last relevant census, that is, 1960, 1970 or 1980 and was terminated upon the diagnosis of first invasive cancer, death, emigration, or on the closing date of the study, 31 December 2000.

## RESULTS

A total of 1 644 958 men gainfully employed in the 1960 census were included in the present study of whom 24 041 developed bladder cancer. Occupations with significantly increased SIRs adjusted either for age and period (‘SIR’) or age, period and social class (‘SEI-adjusted SIR’) in the census of 1960 were listed in Table 1; smoking corrected SIRs (‘Corrected SIR’) were also listed. Adjusting for socioeconomic status caused a small decrease for most occupational groups. For occupations with a significantly increased risk of lung cancer, the smoking-corrected SIRs for bladder cancer decreased and none of the occupations with assumed chemical exposure such as mechanics, iron and metalware workers showed significant results (data not shown). Largely similar findings were found for the 1970 census. Significantly increased SIRs after the smoking correction were observed in the following occupational groups, all without chemical exposure: physicians (SIR 1.29), clerical workers (SIR 1.11), sales agents (SIR 1.10), and administrator and managers (SIR 1.07). Table 2 presents the SIRs of men with the same occupation in two (1960–1970) and three consecutive censuses (1960–1980), respectively, for the groups listed in Table 1. The SIR of physician increased to 1.33 in the two consecutive censuses, and 1.14 in the three consecutive censuses. For chimney sweeps, the SIRs increased to 1.61 and 2.03 in the two and three consecutive censuses, corresponding smoking corrected SIRs to 1.19 and 1.50, but these were not significant.

Similar analyses were carried out for women. The 1970 census was used for women in Table 3 because many more women were recorded as occupationally active in this than in the 1960 census. Only waiters and gardeners had significantly increased SIRs. However, the smoking corrected SIRs did not show significant results. SIRs for women who had the same occupation in different censuses are not shown because of the small numbers. Assistant nurses had significantly increased smoking corrected SIR (1.40, *N*=69, 95% CI 1.09–1.75) in two consecutive censuses, and no significant increase in three consecutive censuses. Smoking corrected SIR was also significantly increased for waitresses (1.41, *N*=41, 95% CI 1.01–1.87) in two consecutive censuses.

## DISCUSSION

Cigarette smoking is the commonest aetiological factor for bladder cancer (Hartge *et al*, 1987, 1993; Harris *et al*, 1990; Andersen *et al*, 1999), but we were unable to distinguish the separate contributions of smoking and occupational exposures because of the absence of smoking information. However, we used a correction factor of 35% of the excess risk of lung cancer as an estimator of smoking risk on bladder cancer. It is possible that in some occupations lung cancer risk may be increased due to carcinogenic exposures, and for these occupations the present correction may be conservative.

Increased SIR after smoking correction was observed for physicians, clerical workers, sale agents and administrators and managers. Our study corroborates the significant association between physicians and bladder cancer reported previously (Shaham *et al*, 1996). Understanding symptoms and an easy access to diagnostic techniques, facilitating diagnosis of relatively benign cancers, may explain some of these elevated risks. On the other hand, a sedentary type of work may be relevant as in clerical workers, and administrators and managers. Physical inactivity might lead to urinary retention and a higher urinary pressure, resulting in more intense and prolonged contact between urine-borne carcinogenic agents and the sensitive basal cells of the distended urothelium (Kunze *et al*, 1992; Mannetje *et al*, 1999).

Although chimney sweeps showed a nonsignificantly increased SIR after smoking correction, the SIRs increased to 1.50 among men with this same occupation was recorded in the three consecutive censuses. A significantly increased SIR of bladder cancer was observed for chimney sweeps in a previous Swedish study (Evanoff *et al*, 1993). The primary exposure of chimney sweeps is soot, which is a complex mixture produced by the combustion of coal, coke, oil and wood, rich in polyaromatic hydrocarbons, a group of compounds with a well-documented carcinogenic effect (IARC, 1983, 1987). Male hairdressers have been classified as an occupational group with probable carcinogenic exposures (IARC, 1993). However, the SIR after our smoking correction did not show significant increase. The later disappearance of increased bladder cancer risks is most probably because use of the relevant carcinogens was discontinued (Czene *et al*, 2003).

In summary, a few occupations were associated with an increased risk of bladder cancer but the effects were modest, even without the correction for smoking. One reason may be poor precision of using occupational titles for exposure, but another is probably the dominant role of smoking over occupational exposures. Sedentary work may be a modest risk factor.

## Figures and Tables

**Table 1:** SIRs of urinary bladder cancer among men in 1960 census

**Occupation**	**Number**	**O**	**SIR^a^**	**95% CI^a^**	**SEI-adjusted SIR^b^**	**95% CI^b^**	**Corrected SIR^c^**	**95% CI^c^**
Physicians	4832	**115**	**1.36**	**1.12–1.61**	**1.29**	**1.06–1.53**	**1.29**	**1.06–1.53**
Administrators and managers	47 422	**995**	**1.13**	**1.06–1.20**	**1.07**	**1.00–1.13**	**1.07**	**1.00–1.13**
Clerical workers	69 060	**1094**	**1.16**	**1.10–1.23**	**1.11**	**1.05–1.18**	**1.11**	**1.05–1.18**
Sales agents	82 553	**1603**	**1.21**	**1.15–1.27**	**1.17**	**1.11–1.23**	**1.10**	**1.04–1.15**
Drivers	109 982	**1615**	**1.07**	**1.02–1.13**	**1.06**	**1.01–1.12**	1.00	0.95–1.04
Mechanics, iron and metalware workers	173 013	**2330**	**1.08**	**1.04–1.13**	**1.08**	**1.03–1.12**	0.99	0.95–1.03
Waiters	2846	**38**	**1.53**	**1.08–2.05**	**1.50**	**1.06–2.01**	1.07	0.75–1.43
Chimney sweeps	1799	**31**	**1.51**	**1.03–2.09**	**1.49**	**1.01–2.06**	1.10	0.75–1.53
Hairdressers	4639	**88**	**1.29**	**1.03–1.57**	**1.26**	**1.01–1.54**	1.10	0.88–1.34
Launderers and dry cleaners	9255	**157**	**1.30**	**1.10–1.51**	**1.27**	**1.08–1.48**	1.13	0.96–1.31
All	1 644 958	24 041	1.00	0.99–1.01	1.00	0.99–1.01	1.00	0.99–1.01

Number, occupationally active person. O, observed cases; bold type, 95% CI does not include 1.00.

a Adjusted for age and period.

b Adjusted for age, period and socioeconomic status.

c Smoking corrected.

**Table 2:** SIRs of urinary bladder cancer for men in the two and three consecutive censuses

**Occupation**	**1960–1970 censuses**					**1960–1970–1980 censuses**				
	**O**	**SEI-adjusted SIR^a^**	**95% CI^a^**	**Corrected SIR^b^**	**95% CI^b^**	**O**	**SEI-adjusted SIR^a^**	**95% CI^a^**	**Corrected SIR^b^**	**95% CI^b^**
Physicians	**95**	**1.33**	**1.07–1.61**	**1.33**	**1.07–1.61**	53	1.14	0.85–1.47	1.14	0.85–1.47
Administrators and managers	414	1.06	**0.96–1.17**	**1.06**	0.96–1.17	118	1.02	0.84–1.21	1.02	0.84–1.21
Clerical workers	**423**	**1.15**	**1.04–1.26**	**1.15**	**1.04–1.26**	133	1.15	0.96–1.35	1.15	0.96–1.35
Sales agents	**744**	**1.16**	**1.08–1.25**	**1.10**	**1.02–1.18**	**272**	**1.17**	1.03–1.31	1.10	0.98–1.24
Drivers	**838**	**1.07**	**1.00–1.14**	1.00	0.93–1.07	305	1.08	0.96–1.21	1.01	0.90–1.13
Mechanics. iron and mctalwarc workers	**1168**	**1.06**	**1.00–1.12**	0.97	0.92–1.0.3	423	1.02	0.93–1.12	0.94	0.85–1.03
Waiters	**19**	**1.82**	**1.09–2.73**	1.30	0.78–1.95	4	1.11	0.29–2.47	0.79	0.21–1.76
Chimney sweeps	**22**	**1.61**	**1.01–2.35**	1.19	0.75–1.74	**14**	**2.03**	1.11–3.23	1.50	0.82–2.39
Hairdressers	62	1.14	**0.88–1.45**	1.00	0.76–1.26	33	1.35	0.9.1–1.84	1.17	0.81–1.60
Launderers and dry cleaners	67	1.16	0.90–1.46	1.03	0.80–1.29	19	0.97	0.58–1.45	0.86	0.51–1.28
All	11 432	1.00	0.98–1.02	1.00	0.98–1.02	4.192	1.00	0.97–1.0.1	1.00	0.97–1.03

O, observed cases; bold type, 95% CI does not include 1.00.

a Adjusted for age, period and socioeconomic status.

b Smoking corrected.

**Table 3:** SIRs of urinary bladder cancer among women in 1970 census

**Occupation**	**Number**	**O**	**SIR^a^**	**95% CI^a^**	**SEI-adjusted SIR^b^**	**95% CI^b^**	**Corrected SIR^c^**	**95% CI^c^**
Gardeners and related workers	11 921	**48**	**1.49**	1.10–1.94	1.06	0.78–1.38	1.06	0.78–1.38
Waitresses	25 228	**131**	**1.28**	1.07–1.51	**1.29**	1.08–1.52	1.19	0.99–1.40
All	1 154 091	3405	1.00	0.97–1.03	1.00	0.97–1.03	1.00	0.97–1.03

Number, occupationally active person; O, observed cases; bold type, 95% CI does not include 1.00.

a Adjusted for age and period.

b Adjusted for age, period and socioeconomic status.

c Smoking corrected.
